# Allosteric activation of the metabolic enzyme GPD1 inhibits bladder cancer growth via the lysoPC-PAFR-TRPV2 axis

**DOI:** 10.1186/s13045-022-01312-5

**Published:** 2022-07-14

**Authors:** Wenlong Zhang, Xin He, Haoli Yin, Wenmin Cao, Tingsheng Lin, Wei Chen, Wenli Diao, Meng Ding, Hao Hu, Wenjing Mo, Qing Zhang, Hongqian Guo

**Affiliations:** 1 Department of Urology, Affiliated Drum Tower Hospital, Medical School of Nanjing University, Institute of Urology, Nanjing University, 321 Zhongshan Road, Nanjing, 210008 Jiangsu China; 2 Department of Urology, Drum Tower Hospital Clinical College of Nanjing Medical University, 321 Zhongshan Road, Nanjing, 210008 Jiangsu China; 3 Department of Urology, Nanjing Drum Tower Hospital, The Affiliated Hospital of Nanjing University of Chinese Medicine, 321 Zhongshan Rd, Nanjing, 210008 Jiangsu China

**Keywords:** Bladder cancer, Metabolic enzyme, GPD1, Tumor suppressor, Allosteric activator

## Abstract

**Background:**

Bladder cancer is the most common malignant tumor of the urinary system. Surgical resection and chemotherapy are the two mainstream treatments for bladder cancer. However, the outcomes are not satisfactory for patients with advanced bladder cancer. There is a need to further explore more effective targeted therapeutic strategies.

**Methods:**

Proteomics were performed to compare protein expression differences between human bladder cancer tissues and adjacent normal tissues. The function of GPD1 on bladder cancer cells were confirmed through in vivo and in vitro assays. Transcriptomics and metabolomics were performed to reveal the underlying mechanisms of GPD1. Virtual screening was used to identify allosteric activator of GPD1.

**Results:**

Here, we used proteomics to find that GPD1 expression was at low levels in bladder cancer tissues. Further investigation showed that GPD1 overexpression significantly promoted apoptosis in bladder cancer cells. Based on transcriptomics and metabolomics, GPD1 promotes Ca^2+^ influx and apoptosis of tumor cells via the lysoPC-PAFR-TRPV2 axis. Finally, we performed a virtual screening to obtain the GPD1 allosteric activator wedelolactone and demonstrated its ability to inhibit bladder tumor growth in vitro and in vivo*.*

**Conclusions:**

This study suggests that GPD1 may act as a novel tumor suppressor in bladder cancer. Pharmacological activation of GPD1 is a potential therapeutic approach for bladder cancer.

**Supplementary Information:**

The online version contains supplementary material available at 10.1186/s13045-022-01312-5.

## Background

Bladder cancer, as one of the most common malignant tumors of the urinary system, ranks 4th in incidence among male malignant tumors [1]. More than 400,000 new cases occur each year worldwide, with over 165,000 deaths associated with bladder cancer [2, 3]. The latest data for 2020 show that 573,000 new bladder cancer cases and 213,000 deaths occurred worldwide [4]. In China, the incidence and mortality rate of bladder cancer are increasing gradually [5]. Based on the depth of bladder wall infiltration, bladder cancer is clinically classified into muscle-invasive bladder cancer (MIBC) and nonmuscle-invasive bladder cancer (NMIBC). Of these, NMIBC is usually multifocal, accounting for approximately 80%, while MIBC accounts for approximately 20% and is characterized by a high incidence of distant metastases even after total cystectomy and systemic chemotherapy, with a 5-year survival rate of less than 5% [6]. Clinical management of bladder cancer has barely progressed in the last 30 years. Surgical resection and chemotherapy are the two mainstream treatments for bladder cancer. Gemcitabine in combination with cisplatin is the first-line chemotherapy regimen for bladder cancer [7]. However, patients with advanced bladder cancer treated with this chemotherapy regimen also had only a 40–60% objective remission rate and only a 5% increase in overall survival [8, 9]. Therefore, understanding the molecular mechanisms underlying bladder cancer progression is essential for exploring more effective targeted therapies.

Glycerol 3-phosphate dehydrogenase 1 (GPD1) catalyzes the conversion of dihydroxyacetone phosphate (DHAP) from glucose and NADH to glycerol-3-phosphate (G3P) and NAD^+^ [10]. The product glycerol-3-phosphate and glycerol is the backbone for lipid biosynthesis [11]. GPD1, together with an isoenzyme in mitochondria named GPD2, facilitates the glycerol-3-phosphate shuttle pathway, which plays an important role in transporting reducing equivalents from the cytoplasm to the mitochondria [12]. GPD1 is widely distributed in tissues, with the highest levels in subcutaneous fat, duodenum and mesenteric fat [13, 14]. The abnormal expression of GPD1 may negatively affect human growth. Mutant GPD1 overexpression causes transient hypertriglyceridemia and fatty liver in infancy [13]. It has been reported that GPD1-deficient mice exhibit enhanced locomotor activity by increasing lipid oxidation [15], while they have reduced obesity and body weight [16]. GPD1 has been identified as a tumor suppressor in breast cancer [17, 18] and exerts antitumor effects in lung and prostate cancers [19, 20]. It has been demonstrated that GPD1 exhibits extremely low expression levels or absence in some tumor tissues [21, 22]. However, GPD1 acts as a brain tumor stem cell marker to promote the progression of glioblastoma [23]. The underlying mechanisms of GPD1 in cancer are still not well studied, especially in human bladder cancer.

Here, we performed proteomics to compare protein expression differences between human bladder cancer tissues and adjacent normal tissues. Many differentially expressed proteins were identified, among which GPD1 expression was significantly decreased in bladder cancer and further validated that GPD1 overexpression showed a suppressive effect on tumor growth in vivo and in vitro, which suggests that GPD1 may be a novel therapeutic target for bladder cancer. Based on virtual screening, we report the identification of wedelolactone (WE), the first reported selective and cell-active activator of GPD1 in our knowledge. WE is a well-characterized allosteric GPD1 activator and a promising small molecule to inhibit bladder cancer growth.

## Methods

### Cell lines and cell culture

5637 and T24 cells were cultured in RPMI 1640 medium, J82 and UMUC3 cells were cultured in DMEM, and SV-HUC-1 cells were cultured in F12K medium. All media were supplemented with 10% fetal bovine serum (FBS), 2 mM l-glutamine, and 100 units/ml penicillin with 100 units/ml streptomycin. All cells were cultured in a humidified atmosphere containing 5% CO_2_.

### Label-free proteomic profiling analysis

All tissues were collected from patients undergoing surgery at the Affiliated Drum Tower Hospital of Medical School of Nanjing University (Nanjing, China). The study was approved by Ethical Committee of Nanjing Drum Tower Hospital, Medical School of Nanjing University with written consent provided by all patients (project number:2018-015-01). Combine the diagnostic imaging features to determine the location of the tumor site. Select intact, erythematous tumors for tumor samples. Take the tissue at least 2 cm away from the tumor site as the normal samples, and the pathologist confirmed that the sample was cancer-free by fast freezing pathology. Tumor samples and para-tumor samples were paired. Additional file 1: Table S1 summarizes the characteristics of the patients. The whole process of label-free proteomics was performed by Jingjie PTM Biolab Co., Ltd. (Hangzhou, China).

### Immunohistochemistry (IHC)

Five-micrometer-thick sections were obtained from paraffin-embedded tissue. Sections were deparaffinized, rehydrated, and endogenous peroxidase activity was eliminated. After washing in PBS buffer (pH 7.4), sections were blocked with 10% goat serum and then incubated with the primary antibody for GPD1(1:200 dilution, 13451-1-AP, Proteintech, China) overnight at 4 °C, followed by incubation with secondary antibodies for 45 min at room temperature according to the manufacturer's instructions. Staining was visualized, and each section was reacted with diaminobenzidine and then counterstained with hematoxylin. Negative control was performed by staining with only secondary antibodies.

### TUNEL assay

Apoptotic cells in tumor tissues were analyzed by terminal deoxynucleotidyl transferase-mediated UTP nick end labeling (TUNEL) staining. Apoptotic cell nuclei were stained with green fluorescence according to the manufacturer's (TUNEL FITC Apoptosis Detection Kit, Vazyme, Jiangsu, China) protocol. Cell nuclei were stained with DAPI.

### Western blotting

Cells were collected, and proteins were extracted by whole cell lysis containing protease and phosphatase inhibitors. Cell debris was removed by centrifugation at 4 °C, and the protein concentration was determined by Pierce BCA assay. Protein content was electrophoresed on 10% SDS–PAGE gels followed by immunoblotting on polyvinylidene difluoride membranes (Biosciences, USA). Antibodies against GPD1 (13451-1-AP, Proteintech) and β-actin (700068, ZenBio) were used.

### RT-PCR

Total RNA from cell or tissue samples was prepared using TRIzol reagent (Invitrogen). Total RNA (1 μg) was reverse transcribed using HiScript Q-RT SuperMix (TaKaRa Biotech). β-actin was used as a normalization gene. Q-PCR assays were performed using a Q-PCR kit (Vazyme Biotech) with QuantStudio 6-Flex (Applied Biosystems, Foster City, CA, USA). The results were expressed as fold change using the comparative threshold method.

### RNA-seq

The cDNA library was constructed by Biomarker Technologies Corporation (Beijing, China). The constructed cDNA libraries were sequenced using an Illumina HiSeq™ sequencing platform. The constructed cDNA libraries of were sequenced on a flow cell using an Illumina HiSeq™ sequencing platform. DESeq and *Q* value were employed and used to evaluate differential gene expression. The false discovery rate (FDR) control method was used to identify the threshold of the *P* value in multiple tests in order to compute the significance of the differences. Here, only gene with an absolute value of log2 ratio ≥ 2 and FDR significance score < 0.01 was used for subsequent analysis. The raw reads were deposited into NCBI Sequence Read Archive (SRA) database with accession number of PRJNA849215.

### Untargeted metabolomics based on ultra-high-performance liquid chromatography quadrupole time of flight mass spectrometry (UHPLC-QTOF-MS)

The LC/MS system was used for metabolomic analysis. Positive ion mode: 0.1% formic acid aqueous solution and 0.1% formic acid acetonitrile. Negative ion mode: 0.1% formic acid aqueous solution and 0.1% formic acid acetonitrile. The raw data were collected using MassLynx V4.2 and then processed by Progenesis QI software based on the Progenesis QI software online METLIN database and Biomark’s self-built library for identification, and at the same time, theoretical fragment identification and mass deviation were all within 100 ppm.

### Flow cytometry staining

5637 cells or T24 cells were incubated with anti-TRPV2 antibody (1:100 dilution, ACC-139, Alomone Labs) at 4 °C for 30 min. Subsequently, cells were washed and incubated with Alexa Fluor™ 594-labeled goat anti-rabbit IgG (H + L) (1:100 dilution, A-11037, Invitrogen, CA, USA) for 1 h. After staining, cells were washed and suspended in PBS for flow cytometric analysis. For detection of intracellular Ca^2+^ concentration in 5637 cells or T24 cells by flow cytometry, Fluo-3 AM (S1056, Beyotime, Jiangsu, China) was used according to the manufacturer's instructions.

### Cell counting kit-8 (CCK-8) assay

Cell viability was analyzed using the CCK-8 assay. A total of 5637 or T24 cells (5000 cells per well) were inoculated in 96-well plates. At the indicated time points, the cells were incubated with 10 μL CCK-8 solution for 2 h at 37 °C, followed by measurement of absorbance at 452 nm. All experiments were performed in triplicate.

### Measurement of G3P and NAD^+^

G3P levels were measured using Glycerol-3-phosphate (G3P) Assay Kit (ab174094, Abcam, Cambridge, MA, USA) according to the manufacturer's instructions. NAD^+^ levels were measured using NAD^+^/NADH Assay Kit (Beyotime, Haimen, Jiangsu, China) according to the manufacturer's instructions.

### Cell apoptosis detection

Apoptotic cells were measured using the membrane-linked protein V/propidium iodide (PI) double staining assay. According to the manufacturer's instructions, 5637 or T24 cells were resuspended in 100 µL of 1× binding buffer and stained with both 5 µL of APC-coupled Annexin V and 5 µL of PI. Cells were then incubated in the dark for 15 min and subjected to flow cytometry analysis.

### Transwell migration assay

The assay was performed using the method described in the previous study [24].

### Tumor sphere formation assay

For the sphere formation assay of 5637 and T24 cells, 5000 cells/well were cultured in serum-free DMEM/F-12 (1:1 ratio) medium supplemented with B-27 (Gibco, USA), 10 ng/mL bFGF, 20 ng/mL EGF, and N2 supplement (Thermo Fisher Scientific, USA) in ultralow attachment 6-well plates to form tumor spheres. Images were collected with light microscopy.

### Colony formation assay

T24 or 5637 cells were seeded into 12-well plates (300 cells per well) and cultured for 10 days. The cells were then fixed in 10% formaldehyde for 15 min and subsequently stained with 1.0% crystal violet for 20 min. The number of colonies formed was counted by ImageJ.

### Cell cycle detection

Cells were collected and fixed in 70% cold ethanol at 4 °C overnight. The ethanol-fixed cells were washed again with PBS, incubated with 50 μg/mL PI containing 20 μg/mL RNase A for 30 min at room temperature, and subsequently analyzed using flow cytometry.

### TRPV2 siRNA interference assay

TRPV2 siRNA sequences were synthesized as follows by GenePharma (Shanghai, China). Synthetic scrambled siRNAs were used as negative controls. Transfections were performed using Lipofectamine 3000 (Invitrogen). Total RNA and proteins were collected 48 h post-transfection.

- siRNA-1: 5′-CCUACAUCCUGCUGCUCAATT-3′;
- siRNA-2: 5′-GGCUGAACCUGCUUUACUATT-3′.

### Tumor models and studies

Six-week-old BALB/c Nude mice were purchased from GemPharmatech Co., Ltd. (Nanjing, China). All animals were assessed to be healthy and free of disease before tumor implantation. Animal study was approved by the Institutional Animal Care Committee of Jiangsu Province and the Ethics Committee of Nanjing Drum Tower Hospital, Medical School of Nanjing University (project number: IACUC-2007015). A total of 5 × 10^6^ T24 cells or 5637 cells were injected subcutaneously into nude mice. Mice were killed after 4 weeks. Tumors were collected, and tumor weights were measured. For WE treatment experiments, vehicle or 50 mg/kg WE was administered intraperitoneally every 3 days starting on day 7 after injection.

### Prediction of potential allosteric sites of GPD1 and virtual screening

The potential allosteric sites were predicted based on the structure of GPD1 (PDB ID:6E8Y) using the Allosite server (http://mdl.shsmu.edu.cn/AST/). Grid-based ligand docking from energetics (GLIDE) software (Schrödinger Maestro 11.4) was used to virtually screen three commercial compound libraries containing over 65,000 compounds to target the predicted sites. The 10 highest scoring compounds were purchased from *MedChemExpress* for experimental use.

### Microscale thermophoresis (MST)

To determine the binding affinity of GPD1 with WE, microscale thermophoresis (MST) was performed using Monolith NT.115 (NanoTemper Technologies GmbH, Munich, Germany) and standard capillaries. GPD1 recombinant protein was purchased from *Proteintech* (Cat. Ag4471), and wedelolactone was purchased from *MedChemExpress* (HY-N0551). The GPD1 recombinant protein was labeled with the fluorescent dye Red-NHS using NanoTemper’s labeling kit MO-L011 Monolith™ according to the manufacturer's instructions. A fluorophore/aptamer ratio of 0.5 was determined by measuring absorbance at 260 and 546 nm using a Nanophotometer™ (Implen GmbH, Munich, Germany). Incubate 200 nM labeled GPD1 protein with 1 µM to 62.5 µM WE. Analysis was performed in binding buffer including 0.05% Tween 20 at 25 °C. The fluorescence signal was normalized, and the Hill equation was fitted using MO Affinity Analysis v2.1.3 software (NanoTemper).

### GPD1 enzyme activity assay

For GPD1 enzyme activity assays, the 100-μL reaction system contained TEA buffer (20 mM), 0.1 mg/mL BSA, 200 μM NADH, 8 mM DHAP, 1 μM GPD1, and compound/DMSO at I = 0.12 (NaCl). The initial rate of DHAP reduction was determined by measuring the change in NADH content via 340 nM absorbance. The absorbance was measured using Tecan infinite 200Pro plate reader (Tecan Ltd., Switzerland).

### Bioinformatics

Gene expression of GPD1 in 31 tumors and their paired normal tissues in the TCGA database were obtained from GEPIA2 (website: gepia2.cancerpku.cn/#analysis).

### Statistical analysis

Data are expressed as the mean ± SEM. When only two value sets were compared, statistical analysis was performed by Student’s *t* test. When the data involved three or more groups, two-way ANOVA was used. Survival analysis was calculated with Kaplan–Meier method, and differences were compared by the log-rank test. *P* < 0.05, *P* < 0.01 or *P* < 0.001 were considered statistically significant and indicated by *, ** or ***, respectively.

## Results

### Proteomics reveals low expression of GPD1 in human bladder cancer tissues

**Fig. 1:**
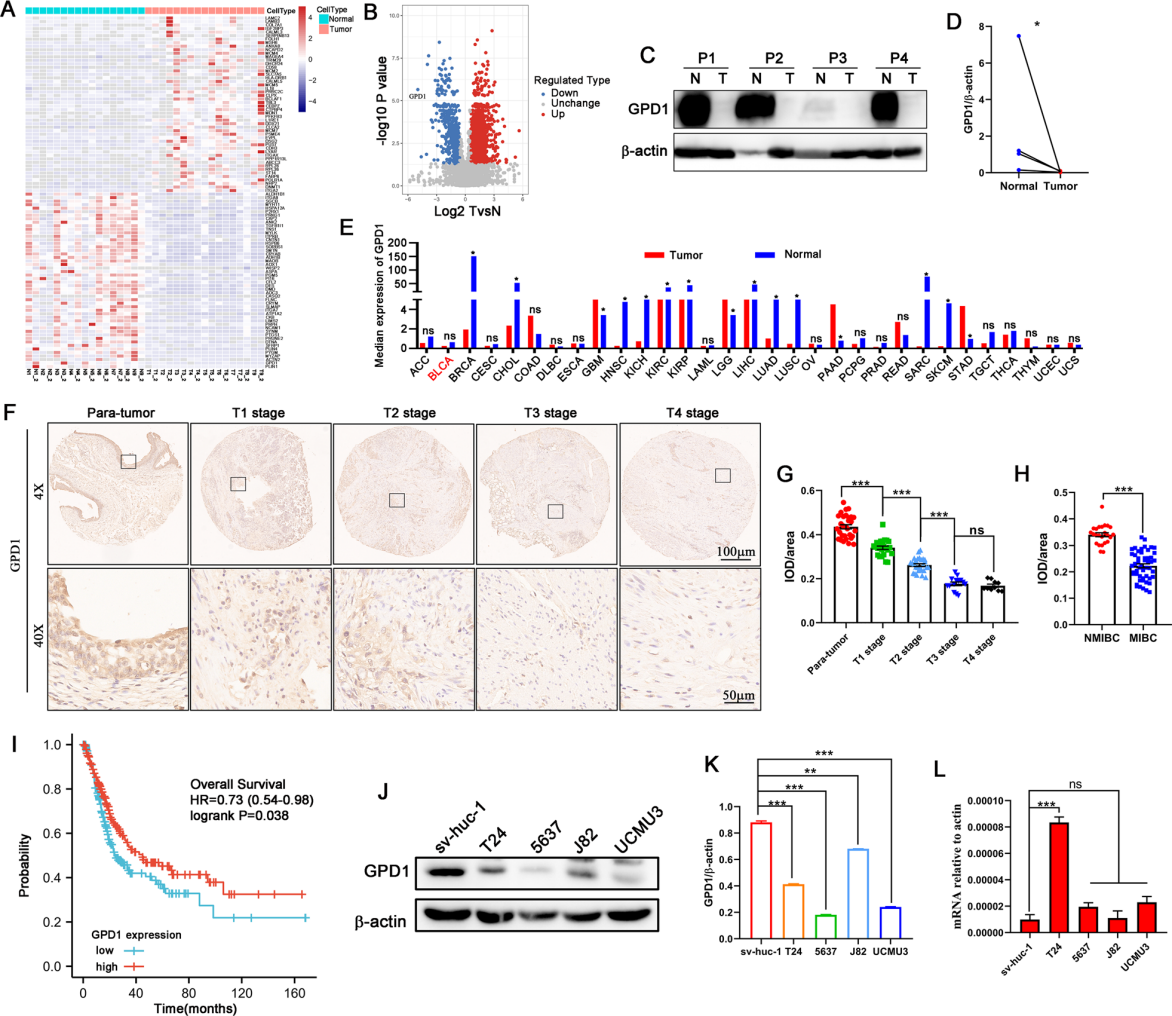
Proteomics reveals low expression of GPD1 in human bladder cancer tissues. **A** The heatmap shows significantly upregulated or downregulated proteins between bladder cancer tissues and matched adjacent normal tissues. **B** Volcano plot of differentially expressed proteins between bladder cancer tissues and matched adjacent normal tissues. **C** and **D** Western blotting to detect GPD1 expression in 4 paired normal and bladder tumor tissue. **E** GPD1 mRNA expression levels in 31 tumors and their paired normal tissues from TCGA database were analyzed by GEPIA tool. **F**–**H** Immunohistochemical examination of GPD1 expression in para-tumor tissue and different stage tumor tissue from patients with bladder cancer. Quantitative evaluation of GPD1 expression represented as IOD/area. **I** Kaplan–Meier plot for GPD1 gene expression to evaluate the probability of overall survival (OS). **J** and **K** Western blotting to detect GPD1 expression in different cell lines. Quantitation of the Western blotting data was performed by ImageJ. **L** RT-qPCR to detect GPD1 mRNA levels in different cell lines

To obtain the protein expression profiles of bladder cancer tissues, we collected 17 pairs of bladder cancer tissues and matched adjacent normal tissues to perform differential expression protein analysis using proteomics. A total of 98,211.0 peptides were identified by profiling, of which 92,295.0 were specific peptides. We identified a total of 8286.0 proteins, of which 6700.0 were quantifiable. With 1.5-fold as the differential expression change threshold and *t* test *P* value < 0.05 as the significance threshold by statistical test, we then identified 1584 proteins with upregulated expression and 518 proteins with downregulated expression in the cancer tissue among the quantified proteins (Fig. 1A and Additional file 2: Table S2). Among these proteins, GPD1 was the most significantly downregulated (Fig. 1B). We detected the expression of GPD1 in 4 pair normal and tumor tissues by western blotting, and the results showed that GPD1 expression was also significantly reduced in tumor tissue (Fig. 1C, D). In the TCGA database, GPD1 mRNA was shown to be at low levels in multiple tumor types, including breast cancer, lung cancer, and prostate cancer. However, no significant differences in GPD1 mRNA levels were observed between normal and tumor tissues in bladder cancer cohort (Fig. 1E). To determine the tissue expression pattern of GPD1 in human bladder tumors, we generated tissue microarrays containing 76 human bladder cancer samples (T1 stage = 24, T2 stage = 28, T3 stage = 14, T4 stage = 10). Staining of these samples using a validated antibody for GPD1 revealed reduced GPD1 expression in tumor tissue compared to normal tissue, and GPD1 expression gradually decreased with bladder cancer progression (Fig. 1F, G). Additionally, GPD1 expression was lower in the MIBC than in the NMIBC (Fig. 1H). In the MIBC cohort, low mRNA levels of GPD1 were also significantly correlated with decreased survival time for bladder cancer patients (Fig. 1I). We further examined the expression level of GPD1 in human bladder cancer cell lines (5637, T24, J82, and UMUC3) and a human urothelial epithelial cell line (SV-HUC-1). Consistent with our analysis of human bladder cancer tissues, the protein levels but not mRNA levels of GPD1 expression were significantly lower in bladder cancer cells than in SV-HUC-1 normal cells (Fig. 1J–L). Together, these results suggest that GPD1 is expressed at low levels in bladder cancer tissues and may have antitumor functions, which prompted us to further explore the role of GPD1 in the development of bladder cancer.

### Overexpression of GPD1 leads to bladder cancer cell apoptosis and inhibits tumor growth

**Fig. 2:**
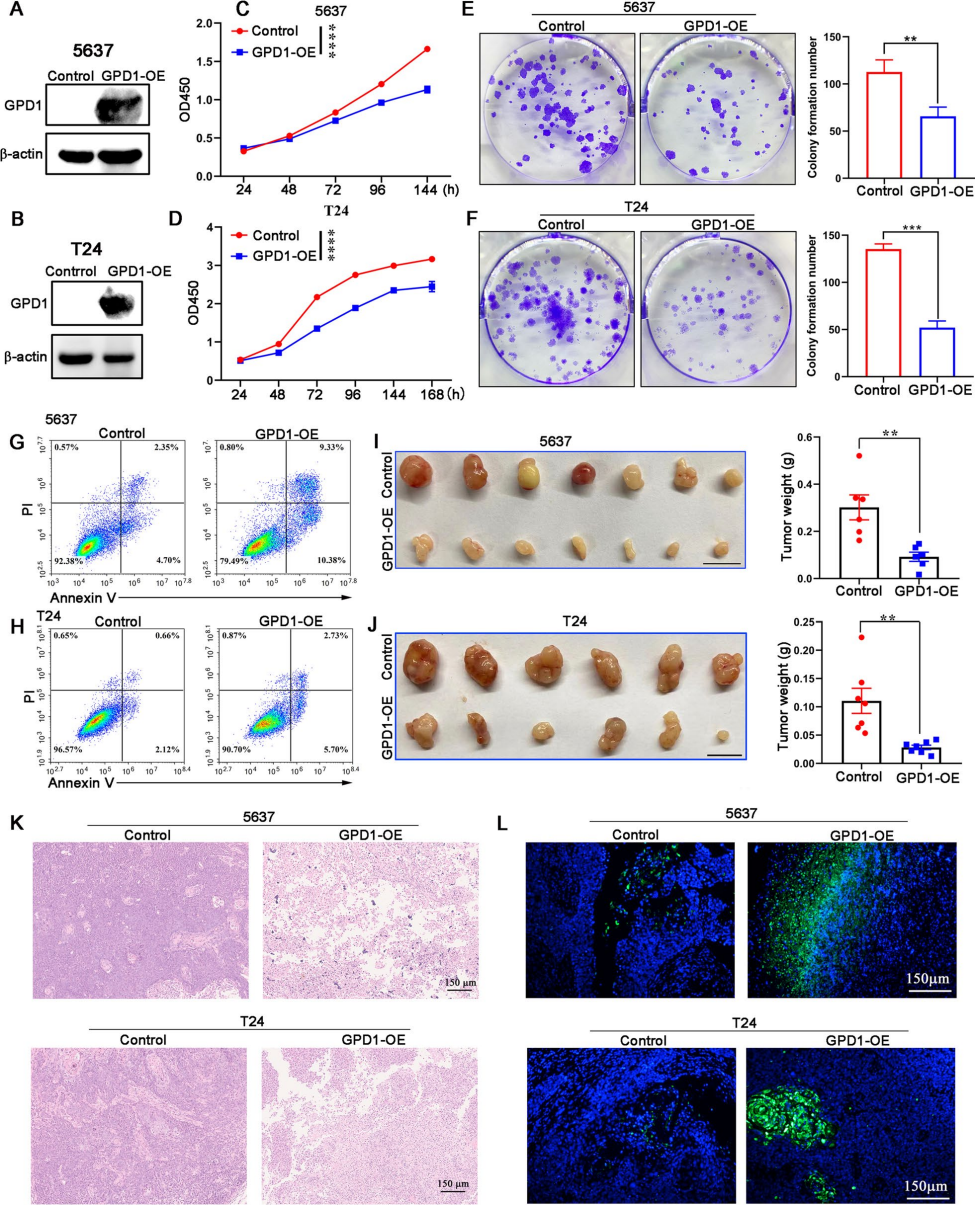
Overexpression of GPD1 leads to bladder cancer cell apoptosis and inhibits tumor growth. **A** and **B** Western blotting to determine GPD1 overexpression in 5637 and T24 cells. **C** and **D** CCK-8 assay to evaluate the proliferation of GPD1-overexpressing 5637 and T24 cells in vitro*.* **E** and **F** Colony formation of 5637 cells (control or GPD1 overexpression) and T24 cells (control or GPD1 overexpression). The number of colonies formed was counted by ImageJ. **G** and **H** Flow cytometry analysis with Annexin V-PI staining was performed to evaluate the percentage of apoptotic cells in GPD1-overexpressing 5637 and T24 cells. **I** Control 5637 cells or GPD1-overexpressing 5637 cells were implanted in BALB/c Nude mice. Tumors were resected and measured 4 weeks later. **J** Control T24 cells or GPD1-overexpressing T24 cells were implanted in BALB/c Nude mice. Tumors were resected and measured 4 weeks later. **K** and **L** H&E staining and TUNEL assay of 5637 tumor tissue or T24 tumor tissue from the control or GPD1 overexpression group

To test our hypothesis that GPD1 may have a cancer cell inhibitory effect, we established bladder cancer cell lines (5637, T24) stably expressing GPD1 via lentivectors transduction. Both constructed cell lines were demonstrated by western blotting (Fig. 2A, B). We observed that GPD1 overexpression could significantly reduce proliferation in both 5637 and T24 cells in vitro by CCK-8 cell assays (Fig. 2C, D) and did not affect the cell cycle (Additional file 5: Fig. S1A, B). The colony formation assay indicated that GPD1 overexpression in 5637 cells and T24 cells significantly decreased the colony number (Fig. 2E, F), additionally supporting the growth inhibitory effect of GPD1 on bladder tumor cells. We further found that GPD1 overexpression significantly promoted apoptosis in 5637 and T24 cells (Fig. 2G, H). Transwell migration and tumor sphere formation assays indicated that the migration (Additional file 5: Fig. S1C, D) and sphere-forming capacities (Additional file 5: Fig. S1E, F) of 5637 and T24 cells were also suppressed by overexpression of GPD1.

To investigate whether overexpression of GPD1 inhibits tumor growth in vivo, 5637 cells and T24 cells stably transfected with GPD1 or control vector were injected subcutaneously into BALB/c Nude mice. Compared with the control group, the GPD1 overexpression group had significantly reduced tumor weight (Fig. 2I, J). We further examined whether GPD1 overexpression was associated with enhanced necrosis or apoptosis of tumor cells in *vivo*. H&E staining and TUNEL assays showed more tumor necrosis and TUNEL-positive cells in tumor tissue from the GPD1 overexpression group (Fig. 2K, L). These results demonstrate that overexpression of GPD1 efficiently inhibits bladder cancer growth.

### GPD1-induced inhibition of bladder cancer depends on its enzymatic activity

**Fig. 3:**
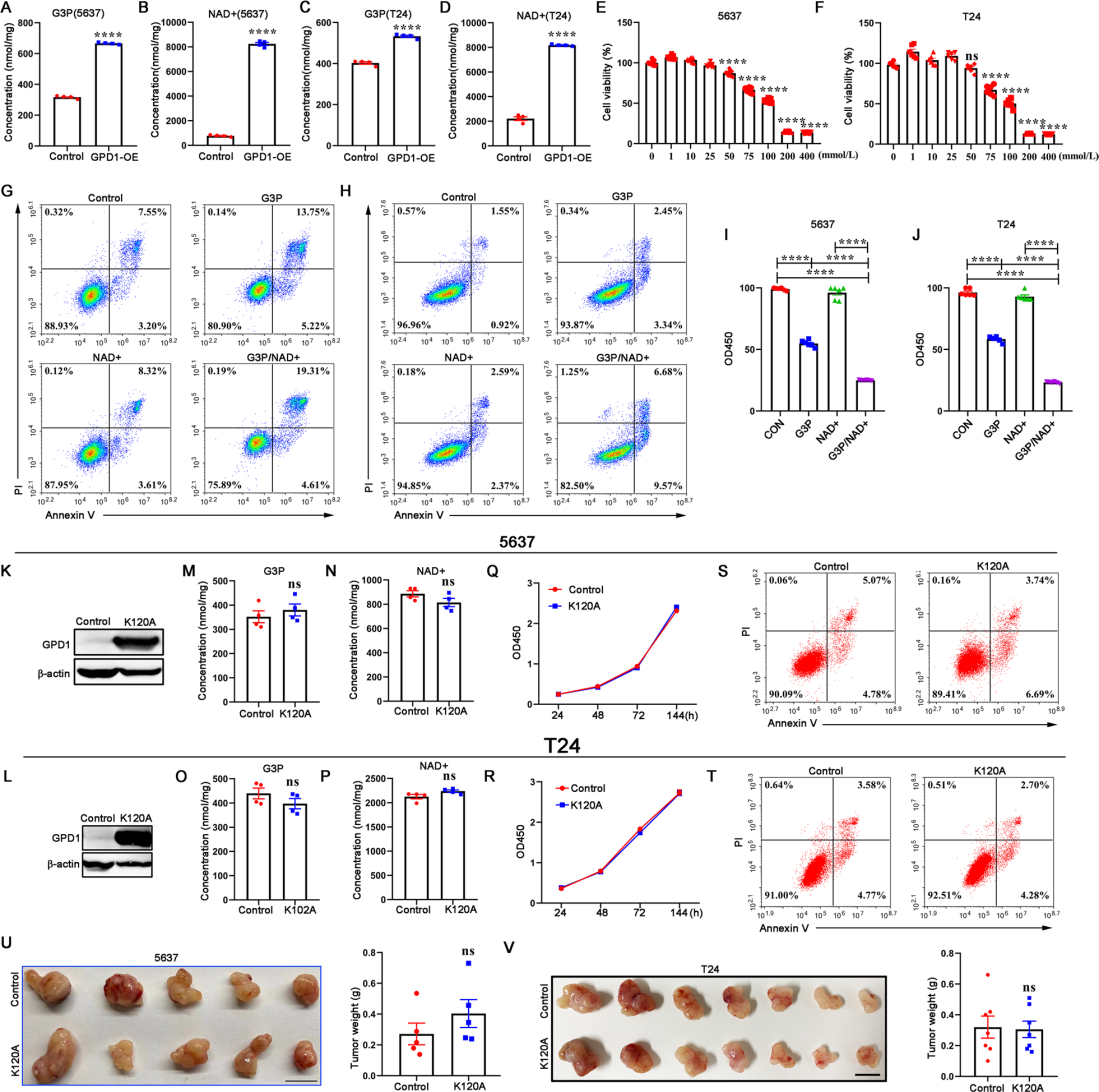
GPD1-induced inhibition of bladder cancer depends on its enzymatic activity. **A** and **B** Detection of intracellular G3P and NAD^+^ levels in control 5637 cells and GPD1-overexpressing 5637 cells. **C** and **D** Detection of intracellular G3P and NAD^+^ levels in control T24 cells and GPD1-overexpressing T24 cells. **E** and **F** CCK-8 assay to evaluate the proliferation of 5637 and T24 cells treated with different doses of G3P. **G** and **H** Flow cytometry analysis with Annexin V-PI staining was performed to evaluate the percentage of apoptotic 5637 cells and T24 cells treated with G3P or NAD^+^. **I** and **J** CCK-8 assay to evaluate the proliferation of 5637 and T24 cells treated with G3P or NAD^+^. **K** and **L** Western blotting to determine K120A GPD1 overexpression in 5637 and T24 cells. **M** and **N** Detection of intracellular G3P and NAD+ levels in control 5637 cells and K120A GPD1-overexpressing 5637 cells. **O** and **P** Detection of intracellular G3P and NAD^+^ levels in control T24 cells and K120A GPD1-overexpressing T24 cells. **Q** and **R** CCK-8 assay to evaluate the proliferation of K120A GPD1-overexpressing 5637 cells and T24 cells in vitro*.* **S** and **T** Flow cytometry analysis with Annexin V-PI staining was performed to evaluate the percentage of apoptotic cells in K120A GPD1-overexpressing 5637 and T24 cells. **U** Control 5637 cells or K120A GPD1-overexpressing 5637 cells were implanted in BALB/c Nude mice. Tumors were resected and measured 4 weeks later. **V** Control T24 cells or K120A GPD1-overexpressing T24 cells were implanted in BALB/c Nude mice. Tumors were resected and measured 4 weeks later

GPD1 is able to catalyze DHAP and NADH into G3P and NAD^+^. In GPD1-overexpressing bladder cancer cell lines (T24 and 5637), we observed an increase in the levels of G3P and NAD^+^ (Fig. 3A–D). Next, we examined whether increasing the concentration of G3P affects bladder cancer cell growth by CCK-8 assay. 5637 and T24 cells were treated with different dosages of G3P. As shown in Fig. 3E and F, the proliferation of 5637 and T24 cells was inhibited by G3P treatment at increasing concentrations. We found that G3P significantly promoted apoptosis in 5637 cells and T24 cells (Fig. 3G, H). Interestingly, the combination of G3P and NAD^+^ further enhanced the pro-apoptotic and anti-proliferative effects (Fig. 3I, J). To further determine that the suppression of tumor growth by GPD1 is dependent on its catalytic activity, which can promote the accumulation of G3P and NAD^+^, we next constructed 5637 and T24 cells overexpressing the GPD1 mutant (K120A variant) (Fig. 3K, L). Mutation of Lys120 in GPD1 led to the loss of catalytic activity [25], which was further evidenced by the fact that K120A GPD1 overexpression did not promote upregulation of G3P and NAD^+^ levels (Fig. 3M–P). As expected, the proliferation and apoptosis of 5637 and T24 cells were not affected by K120A GPD1 overexpression (Fig. 3Q–T). An in vivo study showed that loss of GPD1 catalytic activity did not inhibit bladder cancer growth (Fig. 3U, V). These findings revealed that GPD1 overexpression led to overgeneration of total cellular G3P and NAD^+^, which inhibited bladder cancer growth.

### GPD1 upregulates TRPV2 expression to promote Ca^2+^ influx, leading to apoptosis of bladder tumor cells

**Fig. 4:**
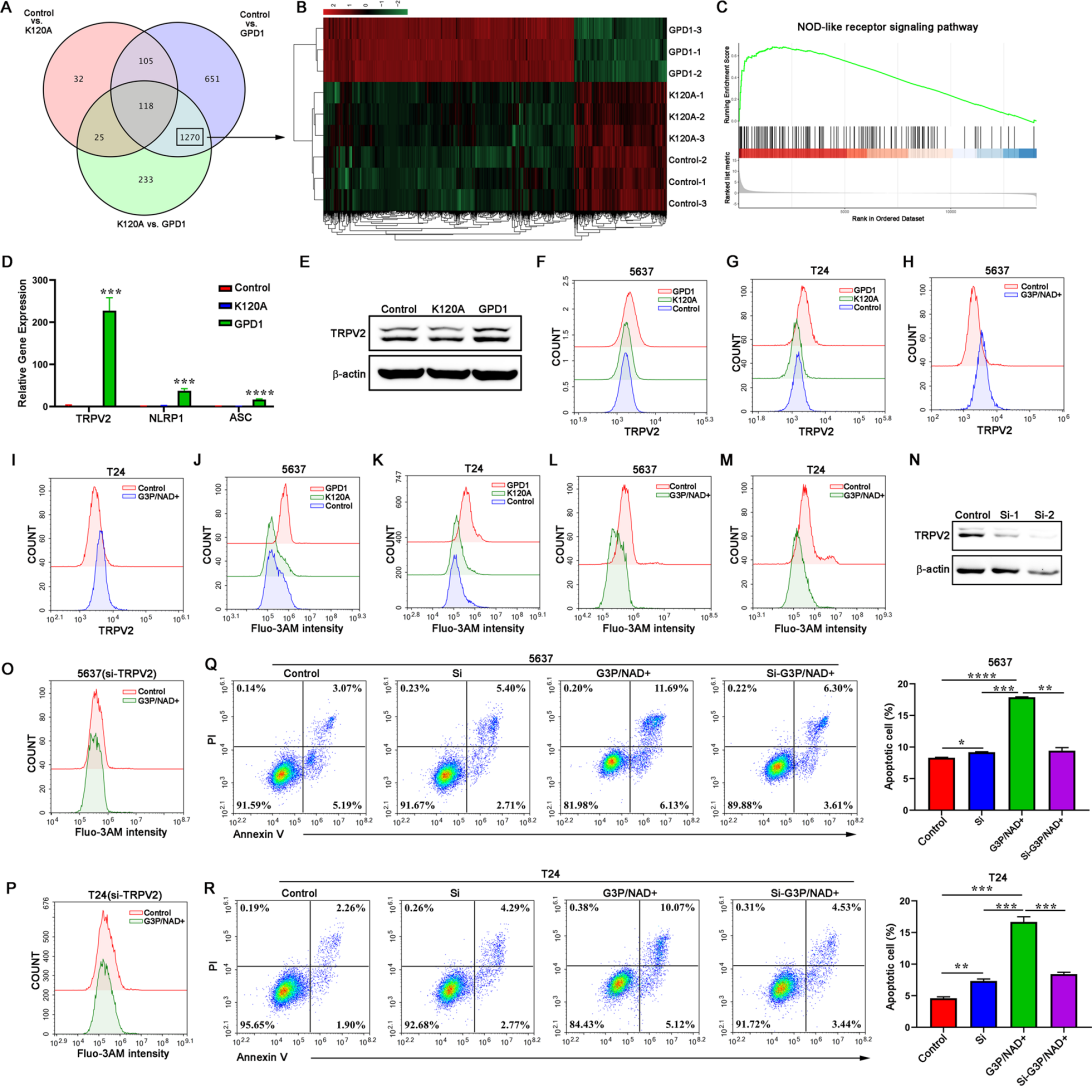
GPD1 upregulates TRPV2 expression to promote Ca^2+^ influx, leading to apoptosis of bladder tumor cells. **A** RNA-seq analysis of control 5637 cells (CON), GPD1-OE 5637 cells (GPD1) and K120A GPD1-OE 5637 cells (K120A). Venn diagram showing the overlap of differentially expressed genes between the three groups. **B** Heatmap showing that the 1270 differentially expressed genes were shared by CON versus GPD1 and K120A versus GPD1. **C** GSEA of the genes associated with the NOD-like receptor signaling pathway. **D** RT–qPCR to validate the mRNA levels of differential genes (TRPV2, NLRP1, and ASC) associated with the NOD-like receptor signaling pathway identified by RNA-seq. **E** Western blotting to detect TRPV2 expression in control 5637 cells (CON), GPD1-OE 5637 cells (GPD1) and K120A GPD1-OE 5637 cells (K120A). **F** and **G** Flow cytometry was performed to detect TRPV2 expression on the membranes of 5637 and T24 cells overexpressing GPD1 or K120A GPD1. **H** and **I** Flow cytometry was performed to detect TRPV2 expression on the membranes of 5637 and T24 cells treated with G3P/NAD^+^. **J** and **K** Flow cytometry was performed to detect intracellular Ca^2+^ levels in 5637 cells and T24 cells overexpressing GPD1 or K120A GPD1. **L** and **M** Flow cytometry was performed to detect intracellular Ca^2+^ levels in 5637 cells and T24 cells treated with G3P/NAD^+^. **N** Western blots show the efficiency of siRNA knockdown of TRPV2 expression in T24 cells. **O** Flow cytometry was performed to detect intracellular Ca^2+^ levels of 5637 cells transfected with siRNAs in the presence or absence of G3P/NAD^+^. **P** Flow cytometry was performed to detect intracellular Ca^2+^ levels of T24 cells transfected with siRNAs in the presence of G3P/NAD^+^. **Q** Flow cytometry analysis of apoptosis in 5637 cells transfected with scramble or siRNAs in the presence or absence of G3P/NAD^+^. **R** Flow cytometry analysis of apoptosis in T24 cells transfected with scramble or siRNAs in the presence or absence of G3P/NAD^+^

To reveal the mechanism underlying GPD1-induced apoptosis of bladder cancer cells, we performed RNA-seq analysis of control 5637 cells, GPD1-OE 5637 cells and K120A GPD1-OE 5637 cells to identify the genes that are affected by GPD1. Over 2144 genes were differentially expressed (fold change > 1.5) between control and GPD1-OE cells, while over 1646 genes were differentially expressed (fold change > 1.5) between K120A GPD1-OE and GPD1-OE cells (Fig. 4A, B and Additional file 3: Table S3). A total of 1270 of the differentially expressed genes (DEGs) were shared by CON versus GPD1-OE and K120A GPD1-OE versus GPD1-OE. Based on KEGG functional annotation and gene set enrichment analysis (GSEA), the 1270 differentially expressed genes were significantly enriched in the NOD-like receptor signaling pathway (Fig. 4C and Additional file 4: Table S4). In this context, the expression of transient receptor potential vanilloid 2 (TRPV2), one of the key genes in the NOD-like receptor signaling pathway, was significantly increased in bladder cancer cells after GPD1 overexpression (Fig. 4D, E). Flow cytometry showed that GPD1 overexpression (Fig. 4F, G) or G3P/NAD^+^ (Fig. 4H, I) upregulated TRPV2 expression on the membranes of 5637 and T24 cells. TRPV2-mediated calcium influx can inhibit cell proliferation and induce apoptosis of bladder cancer cells [26]. We further evaluated the intracellular calcium concentration with a Fluo-3AM probe. The results showed that GPD1 overexpression or G3P/NAD^+^ significantly promoted an increase in intracellular calcium concentration (Fig. 4J–M). To further determine whether GPD1 promotes tumor cell apoptosis via TRPV2-mediated Ca^2+^ influx, we silenced the expression of TRPV2 with siRNA (Fig. 4N). The knockdown of TRPV2 significantly inhibited the elevation of Ca^2+^ concentration (Fig. 4O, P) and reduced apoptosis induced by G3P/NAD^+^ in bladder cancer cells (Fig. 4Q, R). Similar results were observed by using the TRPV2 inhibitor tranilast (Additional file 5: Fig. S2). These data suggest that GPD1 promotes apoptosis in tumor cells through TRPV2-mediated Ca^2+^ influx.

### GPD1 promotes TRPV2 upregulation via lysoPC-PAFR axis

**Fig. 5:**
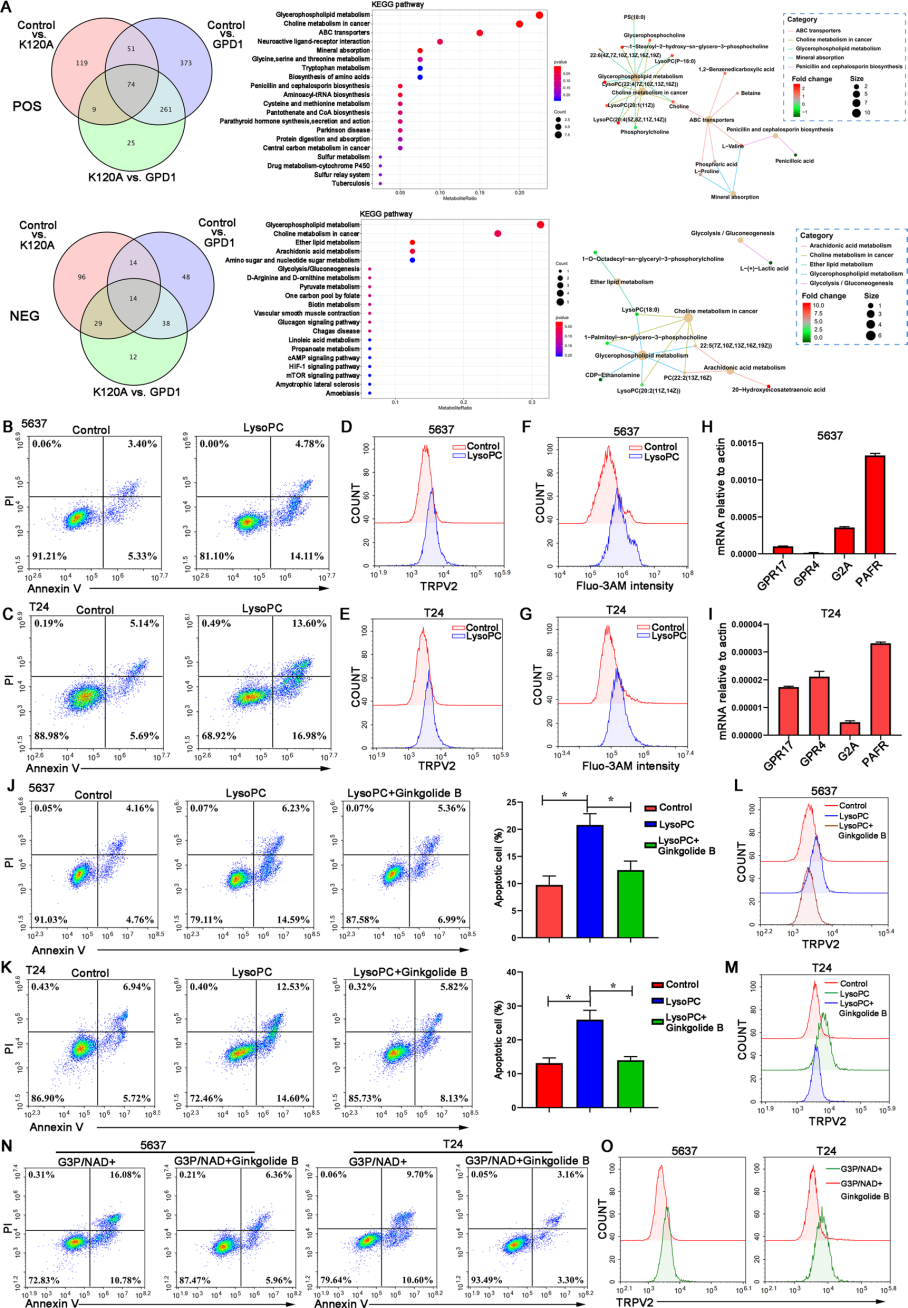
GPD1 promotes TRPV2 upregulation via lysoPC-PAFR axis. **A** Untargeted metabolomics of control 5637 cells, GPD1-OE 5637 cells and K120A GPD1-OE 5637 cells under positive ion mode and negative ion mode. Venn diagram showing the overlap of differential metabolites between the three groups. KEGG was used to explore relevant metabolic pathways. **B** and **C** Flow cytometry analysis of apoptosis in 5637 or T24 cells treated with lysoPC. **D** and **E** Flow cytometry was performed to detect TRPV2 expression on the membranes of 5637 and T24 cells treated with lysoPC. **F** and **G** Flow cytometry was performed to detect intracellular Ca^2+^ levels in 5637 cells and T24 cells treated with lysoPC. **H** and **I** Expression of lysoPC receptors in 5637 cells and T24 cells by RT-qPCR. **J** and **K** Flow cytometry analysis of apoptosis in 5637 or T24 cells treated with lysoPC in the presence of the PAFR inhibitor ginkgolide B. **L** and **M** Flow cytometry was performed to detect TRPV2 expression on the membranes of 5637 and T24 cells treated with lysoPC in the presence of ginkgolide B. **N** Flow cytometry analysis of apoptosis in 5637 cells or T24 cells treated with G3P/NAD^+^ in the presence of ginkgolide B. **O** Flow cytometry was performed to detect TRPV2 expression on the membranes of 5637 and T24 cells treated with G3P/NAD^+^ in the presence of ginkgolide B

To understand how GPD1, as a metabolic enzyme, upregulates the expression of TRPV2, we performed untargeted metabolomics of control 5637 cells, GPD1-OE 5637 cells and K120A GPD1-OE 5637 cells. A total of 261 differential metabolites relevant to GPD1 activity were identified in POS mode, while 38 differential metabolites relevant to GPD1 activity were identified in NEG mode (Fig. 5A). KEGG was used to explore relevant metabolic pathways, among which glycerophospholipid metabolism was the top metabolic pathway regulated in GPD1-OE 5637 cells in both POS and NEG modes. In the glycerophospholipid metabolism pathway, we found that GPD1 overexpression increased the level of lysophosphatidylcholine (lysoPC), which can be converted from G3P (Fig. 5A). We further observed that lysoPC significantly promoted the apoptosis of 5637 and T24 cells (Fig. 5B, C). In addition, similar to GPD1 overexpression, lysoPC upregulated the expression of TRPV2 on the membrane and intracellular Ca^2+^ levels in 5637 and T24 cells (Fig. 5D–G).

To identify receptors for the response to lysoPC, we performed qPCR for all known lysoPC receptors and found the expression of PAFR shared by 5637 and T24 cells (Fig. 5H, I). Immunohistochemical staining indicated that PAFR protein expression in the bladder tumor tissue was elevated compared with the adjacent normal tissues (Additional file 5: Fig. S3A, B). Additionally, GPD1 overexpression had no effect on PAFR expression in 5637 and T24 cells (Additional file 5: Fig. S3C, D). We used ginkgolide B, an inhibitor of PAFR, to confirm that PAFR triggers the response of bladder cancer cells to lysoPC. The results showed that ginkgolide B significantly reduced lysoPC-induced apoptosis (Fig. 5J, K) and TRPV2 expression upregulation (Fig. 5L, M). To further determine that the lysoPC-PAFR axis is necessary for GPD1-induced apoptosis, we evaluated the effect of a PAFR antagonist on apoptosis and TRPV2 expression in 5637 and T24 cells in the presence of G3P/NAD^+^. The results showed that ginkgolide B abrogated G3P/NAD^+^-induced apoptosis (Fig. 5N) and TRPV2 expression upregulation (Fig. 5O). In summary, these data demonstrate that GPD1 regulates TRPV2 expression through the lysoPC-PAFR axis.

### Discovery of a cellularly active GPD1 allosteric activator

**Fig. 6:**
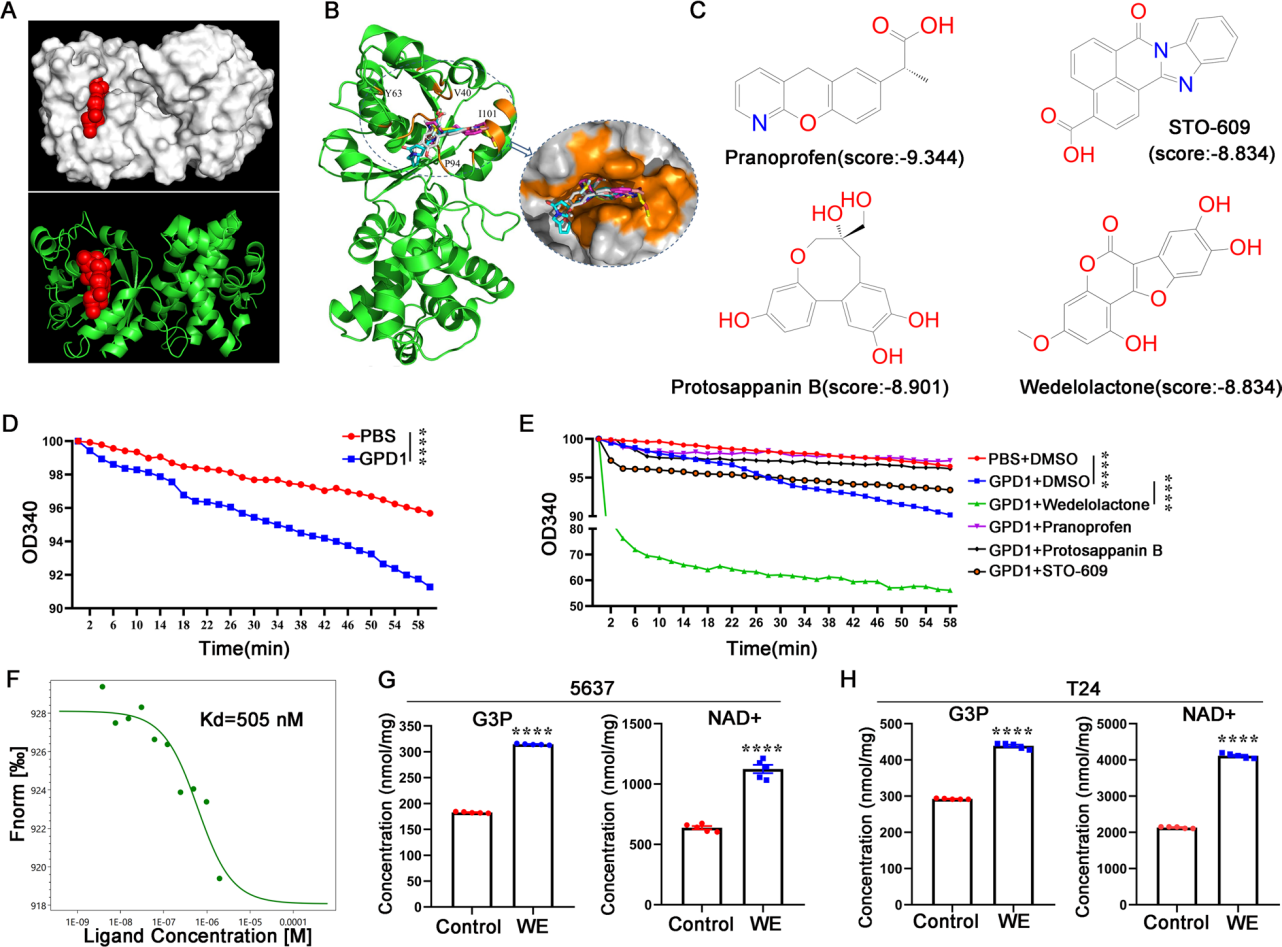
Discovery of a cellularly active GPD1 allosteric activator. **A** Crystal structure of GPD1 protein (PDB ID:6E8Y). The potential allosteric sites were predicted using the Allosite server (http://mdl.shsmu.edu.cn/AST/). Red represents the predicted allosteric sites. **B** Superposition of GPD1 protein with compounds selected by virtual screening. **C.** The structure and docking score of compounds from Bioactive Compound Library Plus on the basis of the top-ranked GPD1-compound binding models. **D** Construction and validation of the GPD1 enzyme activity analysis system. **E** The effect of compounds on the activation of GPD1 enzyme activity was determined by a GPD1 enzyme activity analysis system. **F** Binding affinity of wedelolactone and GPD1 was measured by MST. **G** and **H** Detection of intracellular G3P and NAD^+^ levels in 5637 and T24 cells treated with wedelolactone

Given the antitumor function of GPD1 in bladder cancer, there is considerable interest in discovering activators of the enzyme. First, we hypothesized that functionally critical allosteric sites for GPD1 activation might exist. For identifying these sites, we predicted allosteric sites in GPD1 by using Allosite (http://mdl.shsmu.edu.cn/AST) (Fig. 6A). The predictions indicated that a pocket on the surface of GPD1 may function as an allosteric site, and key residues of the pocket are G10/G12/W39/L50/N60/V92/K100. Next, we virtually docked more than 65,000 compounds into the predicted site (Fig. 6B). Based on the top-ranked GPD1-compound binding models, we selected and purchased 10 compounds (Fig. 6C and Additional file 5: Fig. S4). To evaluate the activity of the compounds on the catalytic activity of GPD1, enzyme activity assays were performed spectrophotometrically at 340 nm. Among the 10 compounds, wedelolactone (WE) from Bioactive Compound Library Plus in *MedChemExpress LLC* had substantial activity in activating the catalytic activity of GPD1 (Fig. 6D, E). We then determined the binding affinity (Kd) between WE and the GPD1 protein by using microscale thermophoresis (MST). The results showed that the binding affinity for WE had a dissociation constant (Kd) of 505 nM (Fig. 6F). To evaluate the activation effect of WE on the catalytic activity of GPD1 in bladder cell lines, we monitored G3P and NAD^+^ levels in the 5637 and T24 cell lines after WE treatment. We found that WE increased both G3P and NAD^+^ at a concentration of 10 µM (Fig. 6G, H). Therefore, WE activates endogenous GPD1 catalytic activity in bladder cancer cells.

### Wedelolactone inhibits bladder cancer growth in vitro and in vivo

**Fig. 7:**
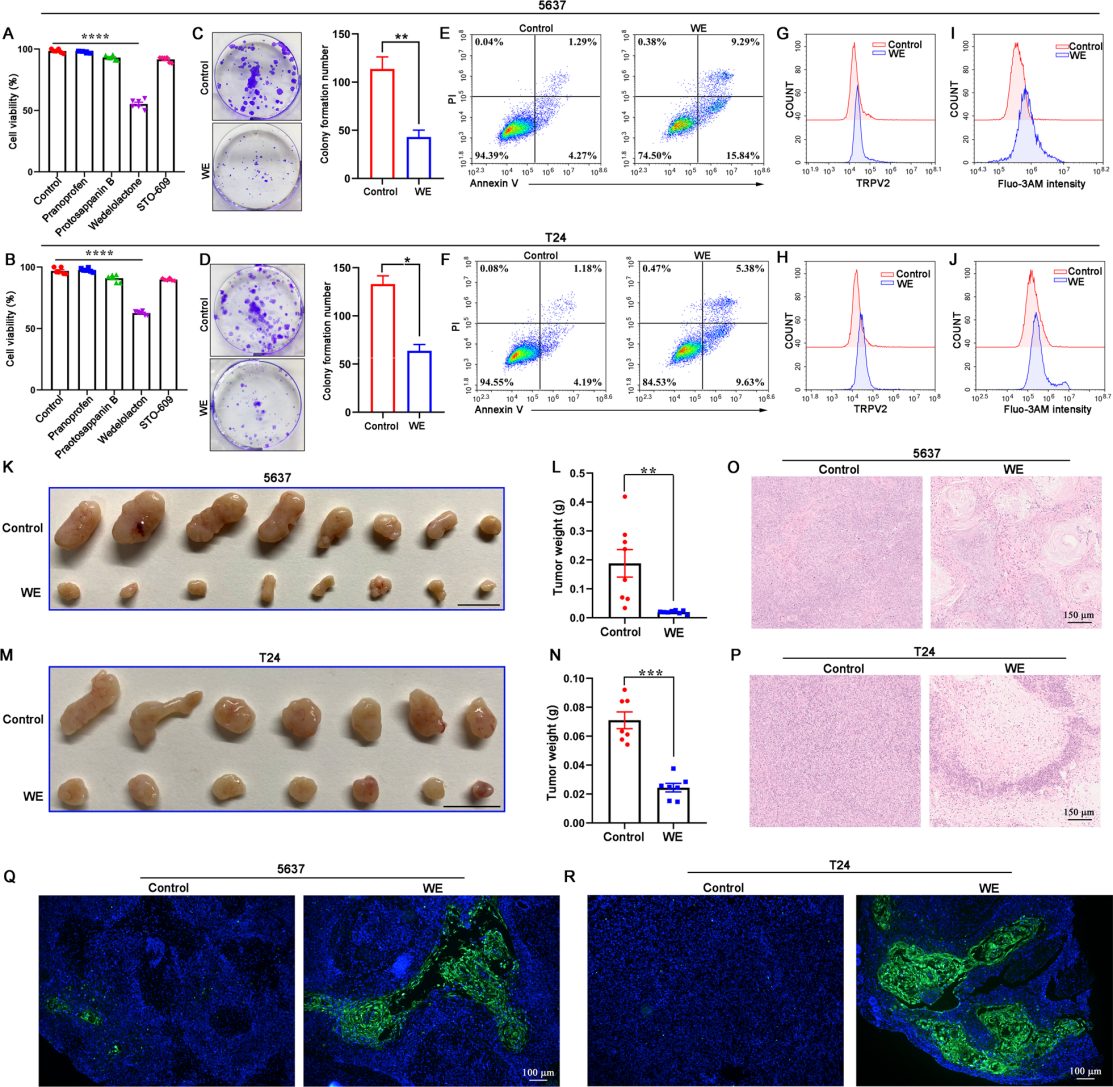
Wedelolactone inhibits bladder cancer growth in vitro and in vivo. **A** and **B** CCK-8 assay to evaluate the proliferation of 5637 and T24 cells treated with different compounds. **C** and **D** Colony formation of 5637 and T24 cells treated with wedelolactone. The number of colonies formed was counted by ImageJ. **E** and **F** Flow cytometry analysis of apoptosis in 5637 and T24 cells treated with wedelolactone. **G** and **H** Flow cytometry was performed to detect TRPV2 expression on the membranes of 5637 and T24 cells treated with wedelolactone. **I** and **J.** Flow cytometry was performed to detect intracellular Ca^2+^ levels in T24 cells treated with wedelolactone. **K** and **L.** Representative photographs of 5637 tumors implanted in mice treated with vehicle or wedelolactone. Tumor weight was analyzed (*n* = 8). **M** and **N** Representative photographs of T24 tumors implanted in mice treated with vehicle or wedelolactone. Tumor weight was analyzed (*n* = 7). **O** and **P** H&E staining of 5637 tumor tissues or T24 tumor tissues from tumor-bearing mice treated with wedelolactone or vehicle. **Q** and **R** TUNEL assay of 5637 tumor tissue or T24 tumor tissue from tumor-bearing mice treated with wedelolactone or vehicle

To further investigate the function of WE in bladder cancer, we assessed the growth and death of bladder cancer cells after WE treatment. Our results showed that WE reduced the proliferation ability of 5637 and T24 cells by CCK-8 cell assay, while other compounds found in virtual screening did not affect proliferation (Fig. 7A, B). The colony formation assay indicated that WE significantly decreased the colony number of 5637 cells and T24 cells (Fig. 7C, D). Additionally, WE induced apoptosis in 5637 cells and T24 cells (Fig. 7E, F). Transwell migration and tumor sphere formation assays indicated that the migration and sphere-forming capacities of bladder cancer cell lines were also suppressed by WE (Additional file 5: Fig. S5). Similar to the effect of GPD1 overexpression, WE upregulated TRPV2 expression (Fig. 7G, H) and intracellular Ca^2+^ concentration (Fig. 7I, J) in 5637 cells and T24 cells. WE is also known as an inhibitor of IKK that is critical for activation of NF-κB. We selected IKK-16, another IKK inhibitor, to confirm that WE promotes apoptosis through GPD1 activation rather than NF-κB inhibition. The results showed that IKK-16 did not promote apoptosis in 5637 and T24 cells and had no effect on G3P and NAD^+^ levels (Additional file 5: Fig. S6). Furthermore, to determine whether WE might suppress bladder cells in vivo, we implanted 5637 cells or T24 cells into BALB/c Nude mice. Compared with the control group, WE treatment significantly reduced tumor weight (Fig. 7K–N). H&E staining and TUNEL assays showed more tumor necrosis (Fig. 7O, P) and TUNEL-positive cells (Fig. 7Q, R) in tumor tissue from the WE treatment group. These results demonstrate that WE efficiently inhibits the growth of bladder cancer in vivo.

## Discussion

In this study, our data show that GPD1 expression is at a low level in bladder cancer. We demonstrate for the first time that GPD1 overexpression can inhibit bladder cancer growth. We found that GPD1 promotes cellular Ca^2+^ influx, leading to apoptosis through the lysoPC-PAFR-TRPV2 signaling axis in bladder cancer cells. Finally, wedelolactone, an allosteric GPD1 activator, was identified for the first time based on virtual screening and was shown to effectively inhibit bladder cancer growth in vitro and in vivo (Additional file 5: Fig. S7).

Over the past 30 years, little progress has been made in the clinical management of bladder cancer. With large-scale gene sequencing efforts, a deeper understanding of bladder cancer biology has been achieved, which has led to the emergence of more favorable targeted therapies in the clinical context. In this study, we compared protein profiles in bladder cancer tissues and adjacent normal tissues by proteomics, from which we found that GPD1 protein levels were significantly downregulated in bladder cancer tissues. Little is known about the role of GPD1 in cancer. GPD1 expression has been found to be low in prostate, lung, and breast cancers [17, 18], and GPD1 was shown to have antitumor effects, which is consistent with our findings and predicts that GPD1 may be a potential therapeutic target in bladder cancer. However, specific expression of GPD1 was observed in brain tumor stem cells and was involved in tumor initiation [23]. Our RNA-seq and metabolomics results suggest that GPD1 can regulate many cellular signaling pathways and metabolic pathways, but these pathways may differ between cancer types. Therefore, the applicability of GPD1 as a target in different tumor types needs to be further investigated.

GPD1 mRNA levels in tumor tissues were not significantly different from those in normal tissues in bladder cancer cohort from TCGA datasets (Fig. 1E). RT-qPCR analysis showed that GPD1 mRNA levels in bladder cancer cells were not lower than in SV-HUC-1 normal cells (Fig. 1L). These data suggest that downregulation of GPD1 protein levels in bladder cancer cells is independent of mRNA transcriptional level alteration. Post-transcriptional regulation is a complex process that encompasses multiple mechanisms. It has been reported that the RNA-binding protein Pub1p reduces GPD1 protein levels and enzymatic activity, but does not alter mRNA levels [27]. We also observed upregulation of several RNA-binding proteins expression in bladder cancer tissues in our proteomics data (Additional file 2: Table S2). This requires further studies to investigate whether these proteins are involved in the downregulation of GPD1 in bladder cancer.

There are few works on the mechanism underlying the role of GPD1 in tumors. Upregulation of G3P levels, an intracellular GPD1 catalytic product, has been found to inhibit tumor cell proliferation in vitro [19]. We have confirmed that the antitumor effect of GPD1 is dependent on the production of G3P by using amino acid mutations to lose GPD1 catalytic activity. Moreover, NAD^+^, another GPD1 catalytic product, was able to enhance the pro-apoptotic effect of G3P. However, the role of G3P in cancer has not been fully investigated. Here, we found that GPD1 activates the NOD-like receptor signaling pathway based on RNA-seq data. Furthermore, we found that an increase in intracellular G3P concentration significantly upregulated TRPV2 expression. Transient receptor potential vanilloid 2 (TRPV2), which belongs to the thermo TRPV subfamily (TRPV1-TRPV4) of transient receptor potential (TRP) channels [28, 29], is a calcium-permeable cation channel. Cannabidiol, an activator of TRPV2, triggers Ca^2+^ influx and apoptosis in the T24 cell line [26], which is consistent with our observations.

We further performed metabolomics and found that overexpression of GPD1 promotes increased levels of lysoPC. A prospective metabolomics study showed that high levels of lysoPC were associated with a reduced incidence of common cancers [30]. In a study of 59 patients with various tumors, reduced lysoPC levels were found to be associated with an increase in inflammatory process parameters (CRP, albumin reduction) and severe weight loss [31]. In our study, lysoPC promoted Ca^2+^ influx through TRPV2 and apoptosis in bladder cancer cells, which was similar to the effect of GPD1 overexpression. A similar study showed that lysoPC induces Ca^2+^ influx and apoptosis through activation of TRPC1/TRPC3 channels in human coronary artery smooth muscle cells [32]. The signaling cell cascade activated by lysoPC is triggered by receptors. The receptors that have been reported are G2A, GPR17, GPR4, and PAFR. We found that lysoPC promotes TRPV2 expression and Ca^2+^ influx via PAFR in 5637 and T24 cells. Overall, this study provides new information that GPD1 induces Ca^2+^ influx and apoptosis through the lysoPC-PAFR-TRPV2 axis.

Based on the potential antitumor function of GPD1, the development of drugs that modulate GPD1 activity may provide a new therapeutic strategy for bladder cancer. Allosteric regulation is a direct, rapid, and widely effective way to regulate protein function, and its dysfunction or abnormal regulation leads to the development of many major human diseases. After more than 50 years of development, the number of allosteric proteins and allosteric regulatory molecules has been increasing, and research on allosteric mechanisms has deepened and gradually translated into drug development and other applications [33]. With the natural advantages of higher target selectivity, fewer side effects, and lower toxicity, the pharmaceutical industry and academia are investing increasing efforts in the development of allosteric drugs [34, 35]. We used the computational chemical biology tool developed in the AlloSteric Database (ASD) to analyze the GPD1 structure and identify potential allosteric regulatory sites. Based on this site, we combined computer-assisted virtual screening and GPD1 catalytic activity assays in vitro to screen and obtain the small-molecule agonist wedelolactone, which can modulate GPD1 catalytic activity, and preliminarily demonstrated its antitumor functions in vitro and in vivo. Wedelolactone, a medicinal plant-derived natural compound, suppresses LPS-induced caspase-11 expression by directly inhibiting the IKK complex [36]. Some studies have also reported that wedelolactone induces caspase-dependent apoptosis in prostate cancer cells via downregulation of PKCε without inhibiting Akt [37]. These studies suggest that wedelolactone may be a multitargeted small-molecule compound beyond targeting GPD1. To improve the target selectivity of wedelolactone, medicinal chemistry optimization is necessary. The combination of treatments may be relatively more effective in eliminating cancer cells with minimal side effects. Metformin, a GPD2 inhibitor, leads to an increase in the concentration of G3P in cancer cells. It has been reported that GPD1 overexpression enhances the anticancer effect of metformin through synergistically increasing G3P levels [19]. This information gives us an insight that the allosteric activators of GPD1 we found in combination with metformin may be a better therapeutic strategy for bladder cancer.

## Conclusion

Taken together, our findings highlight GPD1 as a novel tumor suppressor in bladder cancer. Our data showed that lysoPC converted from the GPD1 catalytic product G3P induced TRPV2 expression via PAFR to promote Ca^2+^ influx, which led to apoptosis of tumor cells. Finally, we screened and obtained the allosteric agonist wedelolactone for GPD1, demonstrating that pharmacological activation of GPD1 is a potential therapeutic approach for bladder cancer.

## Supplementary Information

**Additional file 1: Table S1.** Summary of the characteristics of 17 patients.

**Additional file 2: Table S2.** The proteomic data per sample.

**Additional file 3: Table S3.** The list of DEG identified in RNA-seq.

**Additional file 4: Table S4.** The pathways base on gene set enrichment analysis.

**Additional file 5: Figure S1.** The effect of GPD1 overexpression on tumor cell phenotype. **Figure S2.** Flow cytometry analysis of apoptosis in 5637 cells and T24 cells treated with G3P/NAD+ in presence of tranilast or not. **Figure S3.** GPD1 did not affect the expression of PAFR in 5637 and T24 cells. **Figure S4.** The structure and docking score of compounds on the basis of the top-ranked GPD1–compound binding models. **Figure S5.** The effect of Wedelolactone on tumor cell phenotype. **Figure S6.** The effect of IKK-16 on apoptosis and GPD1 activation in bladder cancer cells. **Figure S7.** Schematic summary.

## Data Availability

The datasets used and/or analyzed during the current study are available from the corresponding author on reasonable request.

## Abbreviations

GPD1: Glycerol 3-phosphate dehydrogenase 1
MIBC: Muscle-invasive bladder cancer
NMIBC: Nonmuscle-invasive bladder cancer
lysoPC: Lysophosphatidylcholine
PAFR: Platelet-activating factor receptor
TRPV2: Transient receptor potential vanilloid 2
WE: Wedelolactone
DHAP: Dihydroxyacetone phosphate
G3P: Glycerol-3-phosphate
IHC: Immunohistochemistry
TUNEL: Terminal deoxynucleotidyl transferase-mediated UTP nick end labeling
UHPLC-QTOF-MS: Ultra-high-performance liquid chromatography quadrupole time of flight mass spectrometry
MST: Microscale thermophoresis
TCGA: The cancer genome atlas
CCK-8: Cell counting kit-8
OE: Overexpression
