# Supplementary material for: Allosteric activation of the metabolic enzyme GPD1 inhibits bladder cancer growth via the lysoPC-PAFR-TRPV2 axis
Source: J Hematol Oncol. 2022 Jul 14;15:93. doi: 10.1186/s13045-022-01312-5 (PMC9284842; doi:10.1186/s13045-022-01312-5)
Supplement: Supplementary file 5 — Additional file 5: Figure S1. The effect of GPD1 overexpression on tumor cell phenotype. Figure S2. Flow cytometry analysis of apoptosis in 5637 cells and T24 cells treated with G3P/NAD+ in presence of tranilast or not. Figure S3. GPD1 did not affect the expression of PAFR in 5637 and T24 cells. Figure S4. The structure and docking score of compounds on the basis of the top-ranked GPD1–compound binding models. Figure S5. The effect of Wedelolactone on tumor cell phenotype. Figure S6. The effect of IKK-16 on apoptosis and GPD1 activation in bladder cancer cells. Figure S7. Schematic summary. [file 13045_2022_1312_MOESM5_ESM.docx]

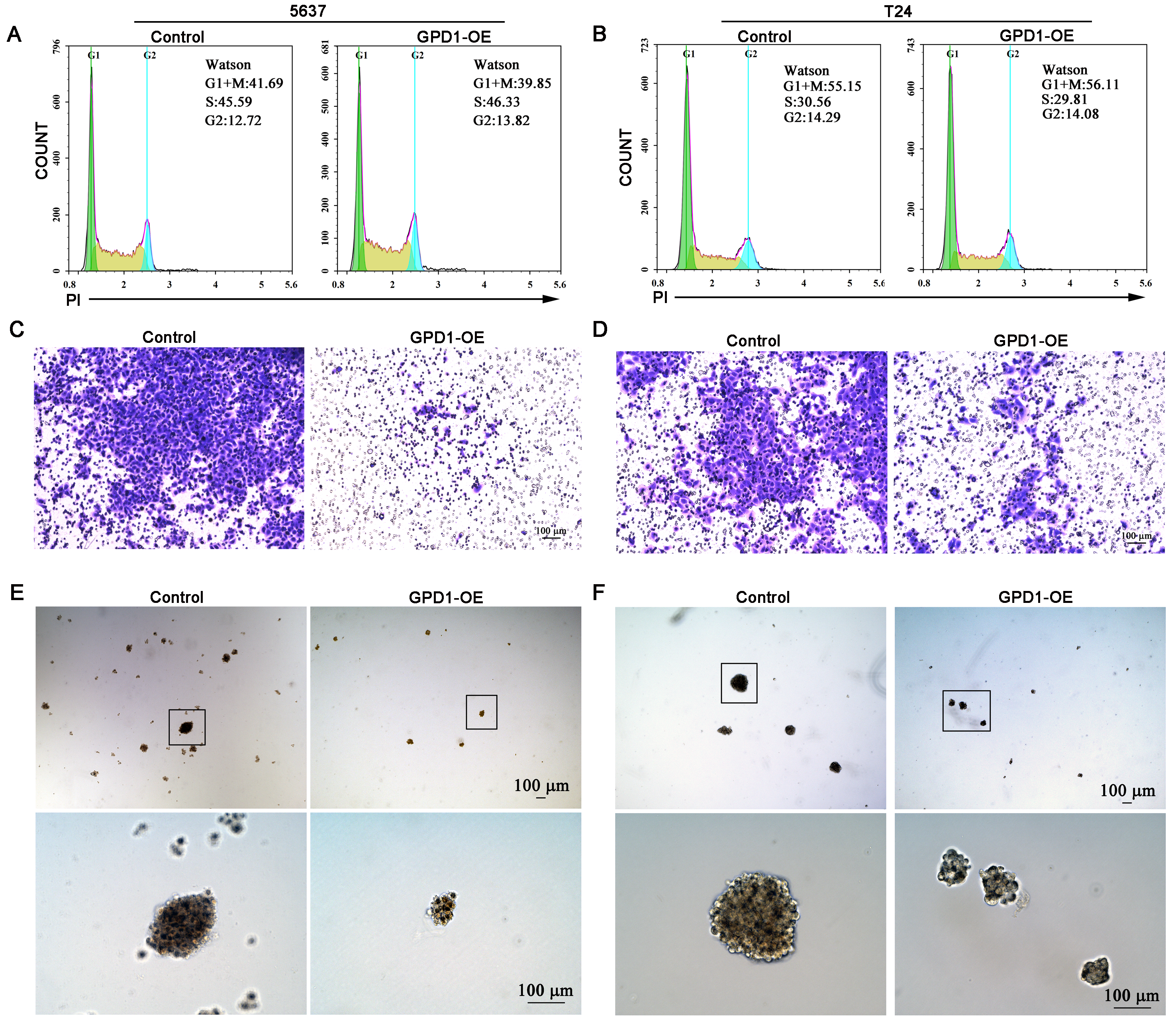


**Figure S1. The effect of GPD1 overexpression on tumor cell phenotype.**

**A-B.** Flow cytometry analysis of cell cycle in 5637 cells (control or GPD1 overexpression) and T24 cells (control or GPD1 overexpression).

**C-D.** Transwell migration ability of 5637 cells (control or GPD1 overexpression) and T24 cells (control or GPD1 overexpression).

**E-F.** Tumor-sphere formation of 5637 cells (control or GPD1 overexpression) and T24 cells (control or GPD1 overexpression).


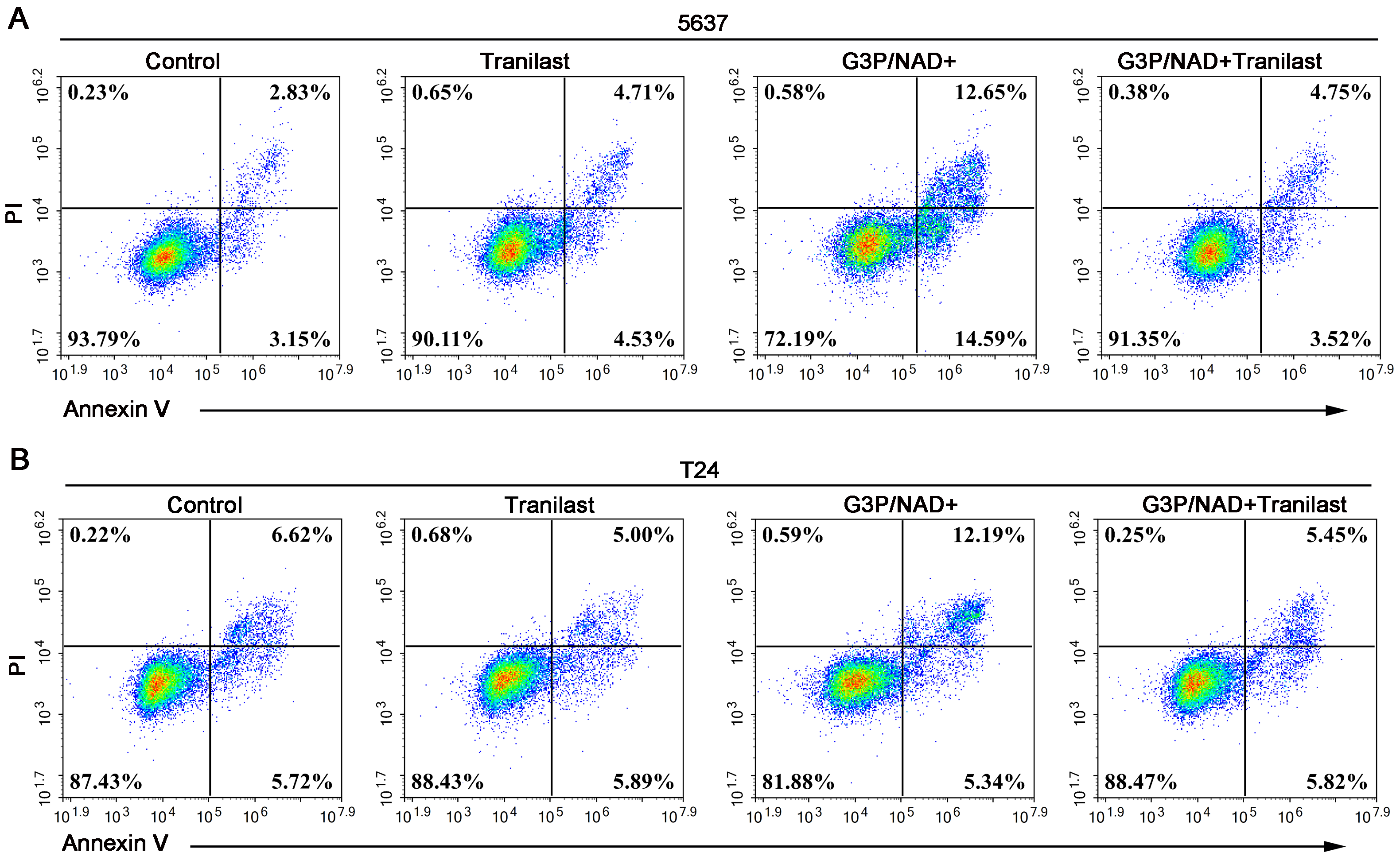


**Figure S2. Flow cytometry analysis of apoptosis in 5637 cells and T24 cells treated with G3P/NAD+ in presence of tranilast or not.**


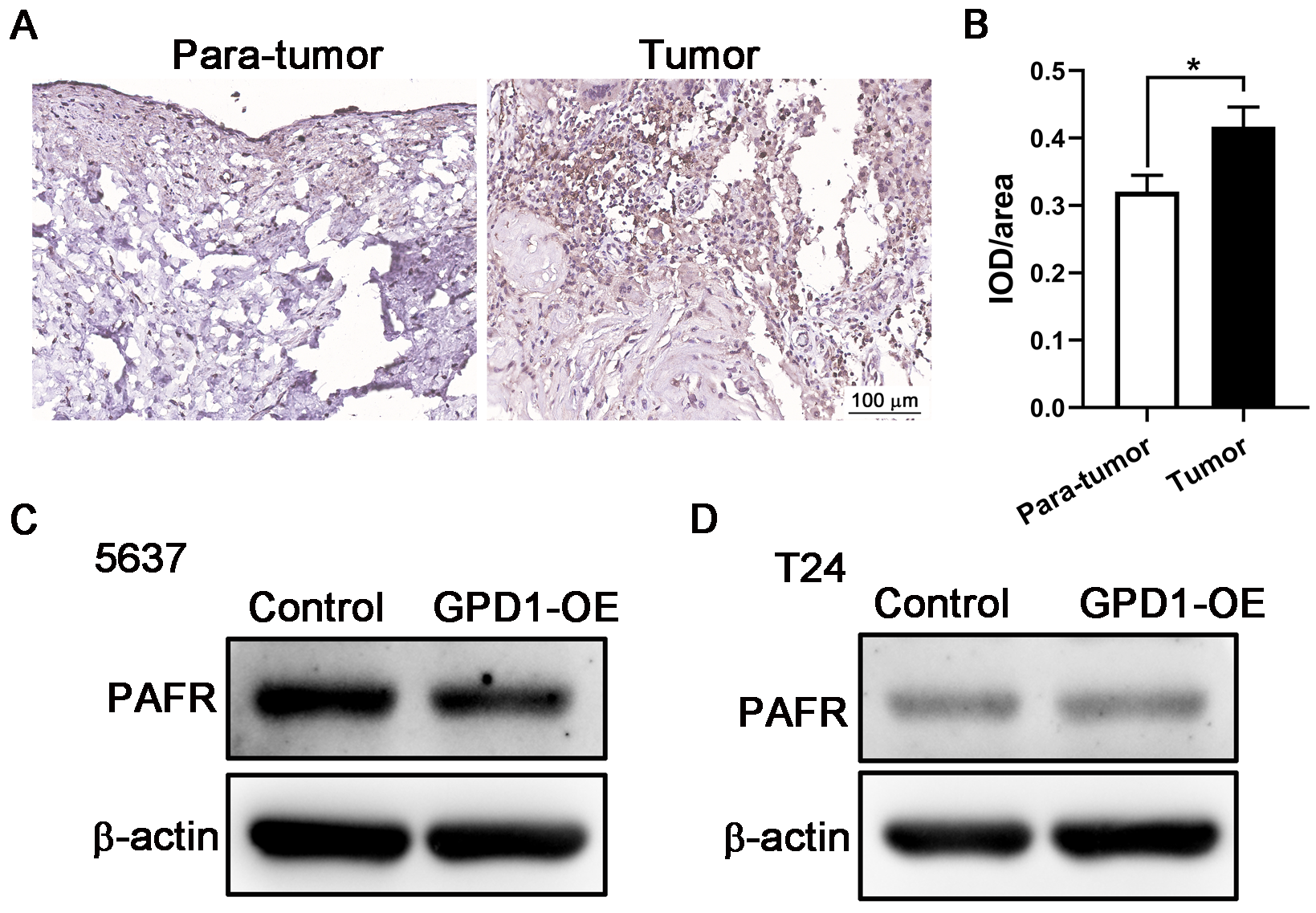


**Figure S3 GPD1 did not affect the expression of PAFR in 5637 and T24 cells.**

**A-B.** Immunohistochemical examination of PAFR expression in para-tumor tissue and tumor tissue from patients with bladder cancer. Quantitative evaluation of PAFR expression represented as IOD/area.

**C.** Western blotting to detect PAFR expression in control 5637 cells and GPD1-overexpressing 5637 cells.

**D.** Western blotting to detect PAFR expression in control T24 cells and GPD1-overexpressing T24 cells.


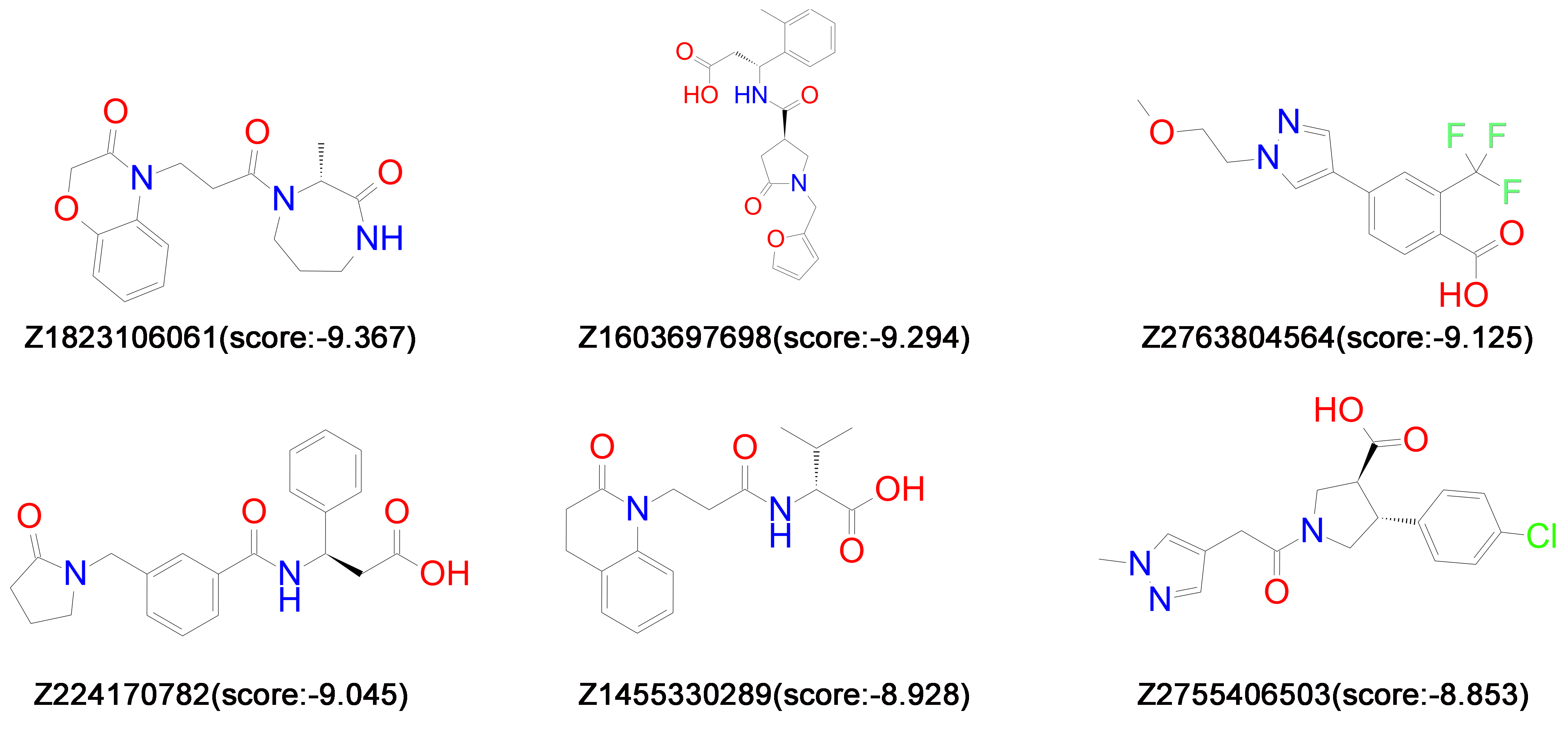


**Figure S4. The structure and docking score of compounds on the basis of the top-ranked GPD1–compound binding models.**


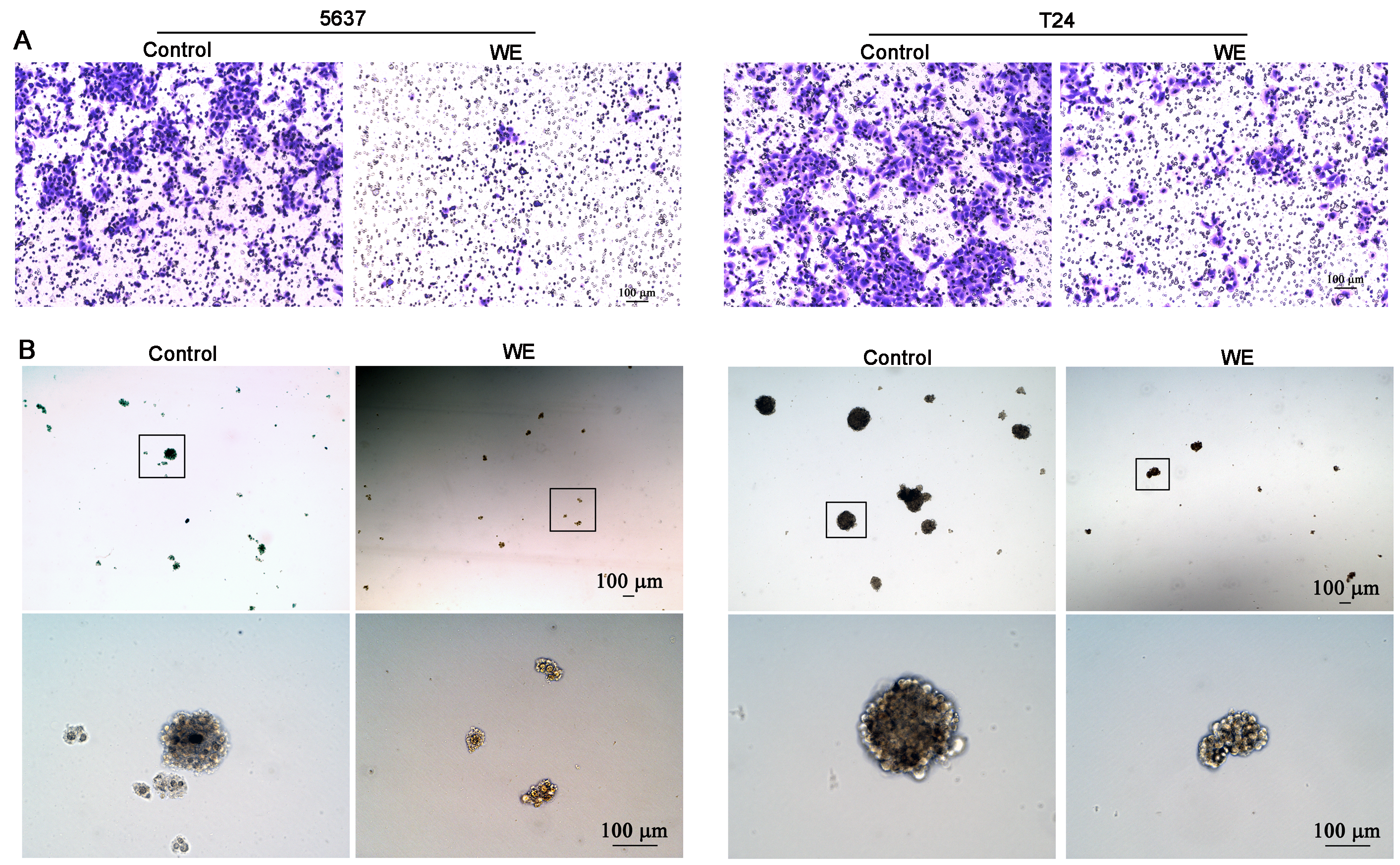


**Figure S5. The effect of Wedelolactone on tumor cell phenotype.**

**A.** Transwell migration ability of 5637 cells and T24 cells treated with Wedelolactone.

**B.** Tumor-sphere formation of 5637 cells and T24 cells treated with Wedelolactone.


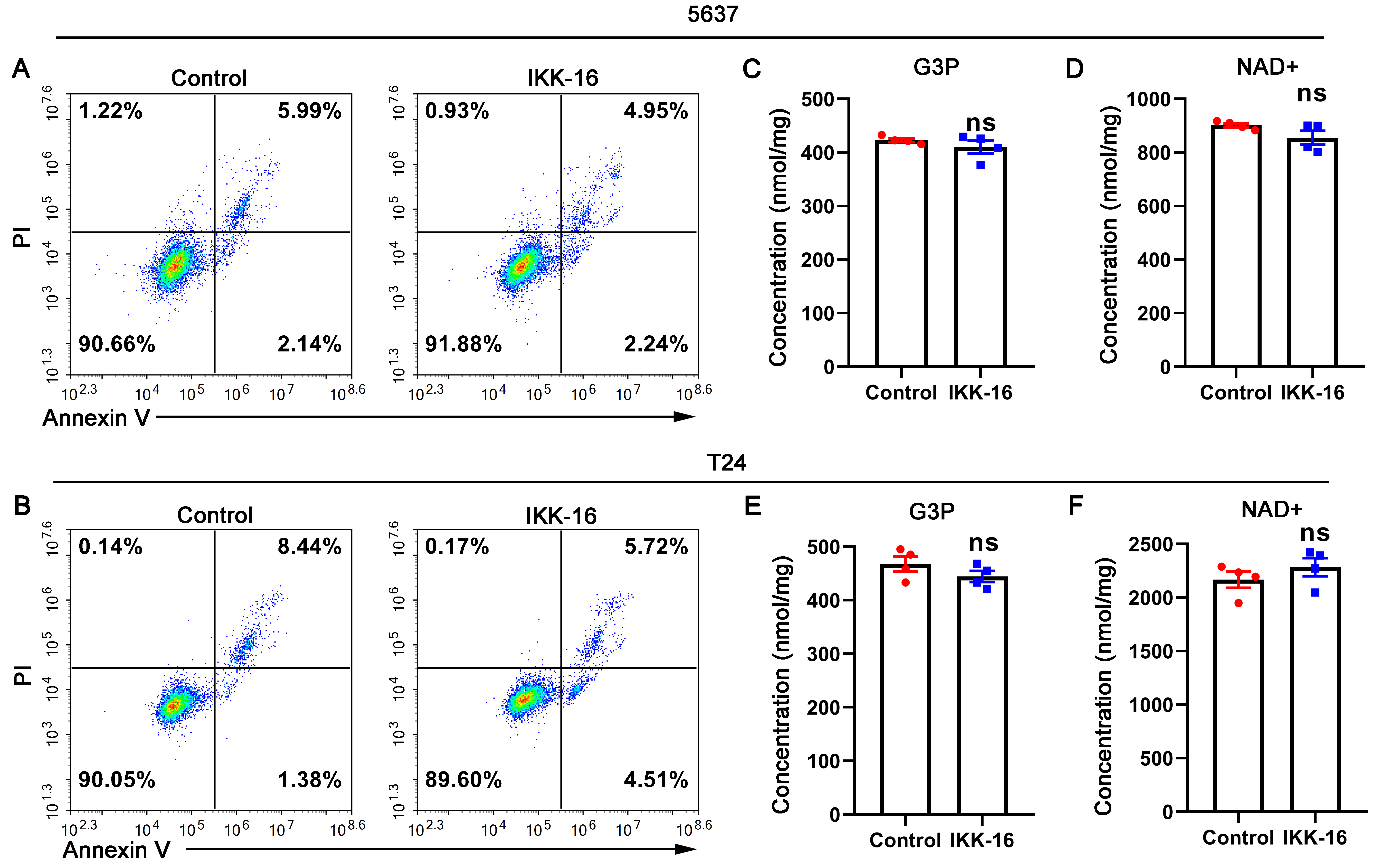


**Figure S6.** **The effect of IKK-16 on apoptosis and GPD1 activation in bladder cancer cells.**

**A-B.** Flow cytometry analysis of apoptosis in 5637 and T24 cells treated with IKK-16.

**C-D.** Detection of intracellular G3P and NAD^+^ levels in 5637 cells treated with IKK-16.

**E-F.** Detection of intracellular G3P and NAD^+^ levels in T24 cells treated with IKK-16.


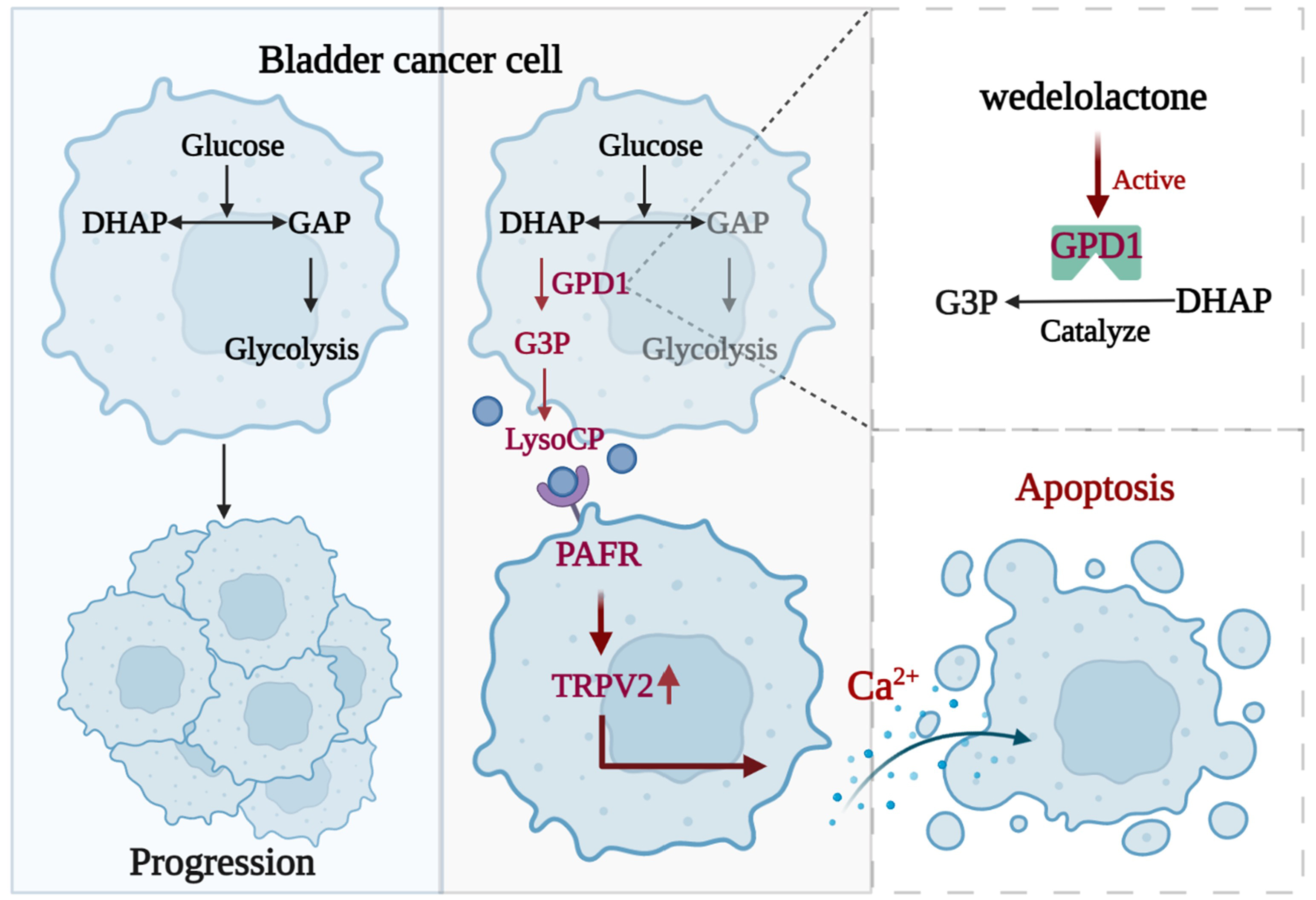


**Figure S7. Schematic summary.**
